# Multifractal analyses of human response time: potential pitfalls in the interpretation of results

**DOI:** 10.3389/fnhum.2014.00523

**Published:** 2014-07-21

**Authors:** Espen A. F. Ihlen

**Affiliations:** Department of Neuroscience, Norwegian University of Science and TechnologyTrondheim, Norway

**Keywords:** response times, 1/*f* noise, multifractal, variability, long-range dependency, fractal

## Introduction

Analyses of response time series have provided insight into mental organization and cognitive processes used in a wide variety of tasks such as simple reaction time, word naming, choice decision, visual search, memory search, and lexical decision (Gilden, 2001). One of the new and frequently used sets of analyses is the numerical definition of scale invariant structure of response time series, also called 1/*f* fluctuations. Component-oriented theories suggest that this scale invariant structure originated from an idiosyncratic mechanism in the cognitive system, whereas interaction-oriented theories argue that scale invariant structure in response time series arises from self-organizing interaction between different sources and mechanisms (cf. Diniz et al., 2011). In this short commentary, new analyses of human response time called multifractal analyses will be introduced, and potential pitfalls of interpreting the results of these analyses will be discussed.

Multifractal analyses quantify the intermittent structure of response time series that are created by interactions between temporal scales of response series (Ihlen and Vereijken, 2010, 2013). Even though these analyses have been recently introduced in analysis of human behavior, their mathematical fundament of these analyses was introduced four decades ago (Yaglom, 1966; Mandelbrot, 1974). Typically, response time series with a large number of trials will contain intermittent periods with a higher number of slow response latencies than the rest of the response series (e.g., Holden et al., 2009). These intermittent periods of slow response latencies might indicate shifts in the participant attention to the stimuli source or active periods of response error corrections (Ihlen and Vereijken, 2010, 2013). In order to quantify the intermittent structure of response time series, multifractal analyses combine two fundamental classes of analyses: (1) model based analyses of the response time distribution and (2) analyses of the dependency of the time ordering of the responses. Class 1 analyses have shown that the response time distributions across cognitive tasks is unimodal, positively skewed, and with a heavy right tail containing the slow response latencies (e.g., Luce, 1986; Holden et al., 2009). Class 2 analyses have shown that the response times have long-range dependency across hundreds and even thousands of trials and, consequently, that the response time series cannot be considered to be independent random variables assumed by class 1 analyses (Gilden, 2001). The long-range dependency (i.e., monofractal structure) of the response time series are numerical, defined as a single scaling exponent by spectral analyses, autocorrelation analyses, detrended fluctuation analysis, and dispersion analysis, to mention but a few (cf. Diniz et al., 2011). However, Class 2 analyses assume that the response time is Gaussian distributed, whereas Class 1 analyses indicate that they have a non-Gaussian heavy tail toward slow response latencies. Multifractal analyses are able to parameterize the non-Gaussian heavy tails that are created using intermittent variation by assessing the complete spectrum of scaling exponents. Thus, multifractal analyses are important extensions of monofractal analyses of response time series.

**Figure 1:**
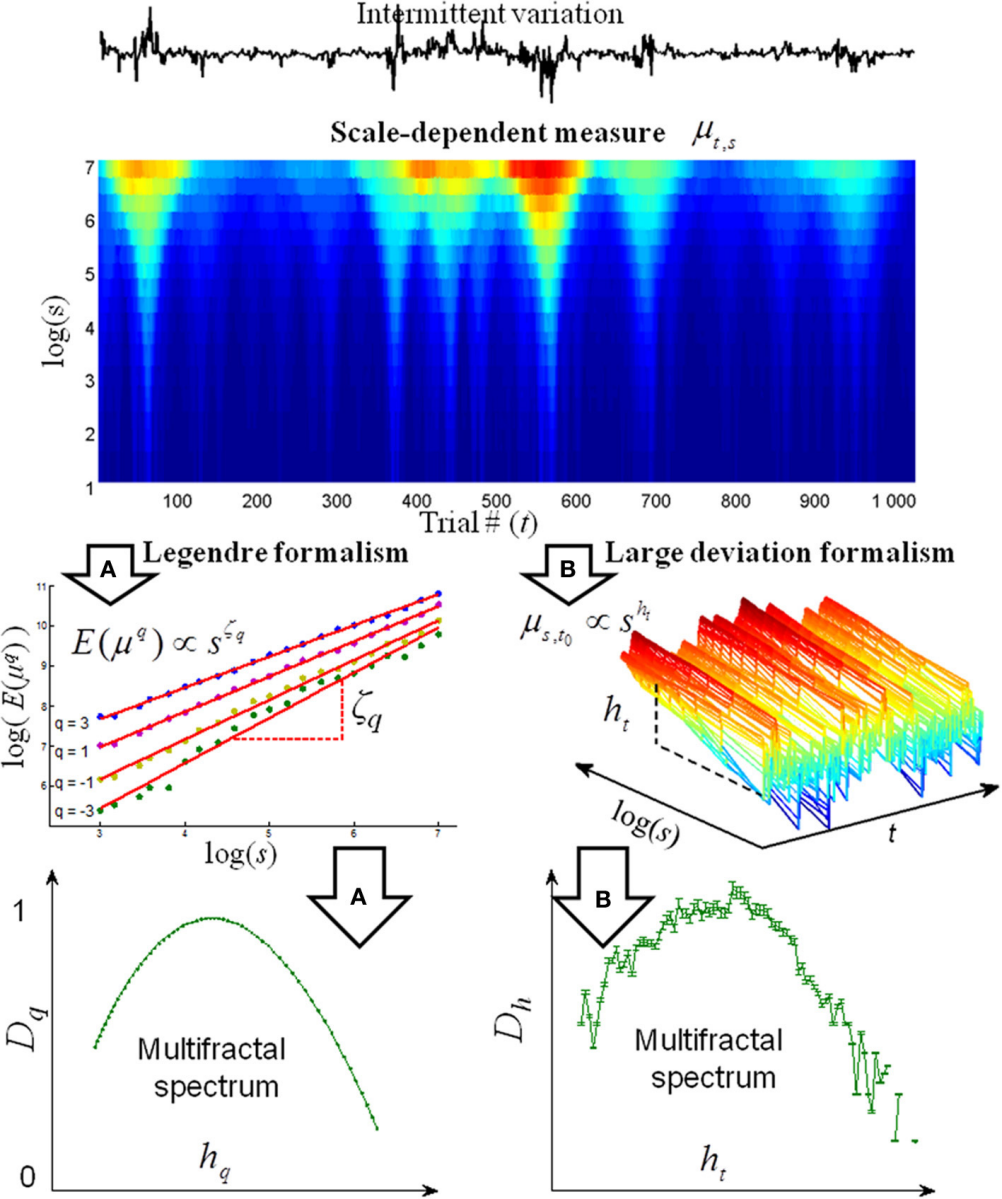
**A flow chart of the estimation of the multifractal spectrum *D*_h_ by analyses within the Legendre formalism (*arrows* A) and large deviation formalism (*arrows* B)**. The basis for all multifractal analyses within both formalisms is the scale-dependent measure (*upper contour plot*) that decomposes the intermittent variation of response time series into both the time and scale domain. The red contours indicate large scale-dependent measures of the response time series that coincide with the time periods of intermittent large variations. In contrast, the blue contours indicate small scale-dependent measures that coincide with the time periods of intermittent small variations. The panel below the top arrow A indicates that the scale-dependent measure is summarized by its *q*-order statistical moment. The statistical moments with positive *q*'s amplify the large *μ_s,t_* (i.e., *red contours*) whereas the statistical moments with negative *q*'s amplify the small *μ_s,t_* (i.e., *blue contours*). The scaling exponent *ζ_q_* numerically defines the power law relation of the intermittent periods with large (i.e., positive *q*'s) and small variation (i.e., negative *q*'s). The panel below the bottom arrow A illustrates a multifractal spectrum *D_h_* estimated from *ζ_q_*. The panel below the top arrow B illustrates the direct estimation of the local singularity exponent *h_t_* as the local slope of log(*μ_s,t_*) vs. log(*s*) for each time instant *t*. The panel below the bottom arrow B illustrates the multifractal spectrum *D_h_* estimated from the distribution of local singularity exponent *h_t_*. Adapted from Ihlen and Vereijken (2013).

All multifractal analyses are based on a decomposition of the response time series into a scale-dependent measure that identifies the periods of intermittent variation (see upper panel of Figure 1). The scale dependent measure is the basis for computation of the multifractal spectra along two formalisms (see arrows A and B in Figure 1). In the Legendre formalism, the scale-dependent measure *μ_s,t_* is used in the computation of the *q*-order moment. *μ_s,t_* is amplified by the positive *q*-orders in the periods with large variation, whereas *μ_s,t_* is amplified by the negative *q*-orders in periods with small variation. An exponent ζ_*q*_ is then estimated from the scaling of each of the *q*-order moments before the multifractal spectra are computed from *ζ_q_* (see Ihlen and Vereijken, 2013 for further details). In the large deviation formalism, local exponents are computed from the scale-dependent measure *μ_s,t_*, and the multifractal spectrum is estimated from the distribution of the local exponents. The increased width of multifractal spectra will reflect more distinct periods of intermittent variation in response time series (see example in Figure 2 in Ihlen and Vereijken, 2013). Additional surrogate tests also detect the periods influenced by multiplicative interactions between temporal scales (Ihlen and Vereijken, 2010). The different multifractal analyses like *structure function approach, entropy analyses, wavelet transformation modulus maxima, gradient modulus wavelet projection*, and *multifractal detrended fluctuation analysis* are defined by the particular way the scale-dependent measures are computed (Ihlen, 2013a; Ihlen and Vereijken, 2013). The Legendre and large deviation formalisms contain statistical assessments of multifractality. Various geometrical assessments have been suggested in the literature that estimates the box counting dimension of the time series (e.g., Russel et al., 1980; Chaudhuri and Sarkar, 1995). However, these methods are only numerically stable for positive *q* orders and, consequently, only estimate the left tail of the multifractal spectrum. Technical details for the computation of different multifractal analyses within the Legendre and large deviation formalisms, their parameter settings, Matlab codes, and comparison of their performance can be found elsewhere (Kantelhardt et al., 2002; Turiel et al., 2006; Kantelhardt, 2011; Ihlen, 2013a). Multifractal analyses have been applied to several cognitive tasks like simple reaction time, word naming, choice decision, and feedback manipulation (Ihlen and Vereijken, 2010; Kuznetsov and Wallot, 2011). All results from these studies indicate that response time series have multifractal properties that are not described by conventional monofractal analyses and that some of these properties might be task dependent.

## Potential pitfalls in the interpretation of multifractal analyses

The interpretation of multifractal spectra of response time series has potential pitfalls. First, the multifractal spectra alone do not indicate that intermittent response time variation is generated by interaction between temporal scales. Wide multifractal spectra of response time series can reflect a power-law response time distribution and not intermittency generated by multiplicative interactions (Ihlen, 2013b). Surrogate tests have to be used to properly identify multiplicative interactions between temporal scales. In these tests, surrogate versions of the response time series are created that eliminate the interaction between temporal scales but preserve all other statistical properties. Multiplicative interaction is present when there is a significant difference between response time series and its surrogate series (e.g., Ihlen and Vereijken, 2010).

Second, response time series of 1000 trials might be too small to establish the presence of multifractality. An ideal monofractal signal will have an infinite number of scales whereas the 1000 trials of response series will only give three scales of order (i.e., 10, 100, and 1000 trials). However, in contrast to ideal monofractal signal, a multifractal signal has scale invariant properties only up to a maximum scale (Bacry et al., 2001). The *q*-order moments and scale-dependent measure converge into a single point on this maximum scale. Thus, in contrast to monofractal analyses, it is sufficient for multifractal analyses to include scales up to the maximum order. Assuming that the signal originates from a prototypical multifractal process, called a multiplicative cascade, the maximum scale could be assessed by analysis of the autocorrelation function (Bacry et al., 2001). Nevertheless, the estimation error of the multifractal spectra related to the number of trials in the response will also be dependent on the chosen *q*-range for the methods within the Legendre formalism and the unknown degree of multifractality. Large degree of multifractality will need large number of trials for a robust assessment of the tails of the multifractal spectra. Consequently, multifractal analysis is quite sensitive to differentiate between monofractal and multifractal response time series, but not between response time series with large degree of multifractality. Furthermore, multifractal analysis of moderately sized response time series will both be more susceptible to noise and non-stationarities compared to longer time series (Ihlen, 2013a). A possible solution is to compare the results of two or more multifractal analysis before interpreting the results. Large deviations in the results of two multifractal analyses indicate that response time series deviate from multifractality and that the results from these analyses must be interpreted with caution.

Third, no single multifractal analysis seems to have superior performance assessing the multifractal spectra of response time series. Previous studies statistical methods based on wavelet transformation, like wavelet transform modulus maxima, has been shown to superior to conventional methods based on the structure function (Muzy et al., 1993). Furthermore, both multifractal detrended fluctuation analysis and gradient modulus wavelet projection has shown superior performance to wavelet transform modulus maxima on moderate sized time series (Kantelhardt et al., 2002; Oświkęcimka et al., 2006; Turiel et al., 2006). Kelty-Stephen et al. (2013) have suggested that an entropy based analysis is the best method to assess the multifractal spectrum from response time series and that other multifractal analyses have inferior performance compared to this method using their choice of a scale-dependent measure. However, recent systematic comparison of multifractal analyses shows that all multifractal analyses have different pros and cons and that no single analyses seem to be superior to others (Ihlen, 2013a).

Fourth, the origin of multifractal and intermittent variation in response time series is still debated. Intermittent variation in response time has been suggested to be caused by changes in the participants' attention to stimuli or intermittent error corrections (Ihlen and Vereijken, 2010) and linked to cognitive phenomena like strong anticipation (Stephen and Dixon, 2011). Furthermore, multifractal spectra have been suggested to reflect to a greater extent the presence of self-organization and interaction-dominant dynamics compared to the outcomes of conventional monofractal analyses (Ihlen and Vereijken, 2010; Kelty-Stephen et al., 2013). The interaction-dominant view has been suggested to contrast explicit models of an idiosyncratic mechanism in the cognitive system specific to cognitive tasks or the dynamics of particular localized components (e.g., Van Orden et al., 2003). However, idiosyncratic mechanisms for multifractal variations have been suggested for human locomotion and cardiac function, which indicates that intermittent variations can be generated by task specific components (Ivanov et al., 1998; West and Scafetta, 2003). It is unlikely that any analysis or model will provide conclusive evidence on the generating processes of multifractal variation in response time series (Hasselman, 2013; cf. Kantz and Schreiber, 2004). The generating processes of multifractal and intermittent variation should be decided by experimentation under conditions of strong inference (Hasselman, 2013). Consequently, experimental design should be use to confirm predicted changes in the multifractal spectra. Predicted covariation between local scaling exponents of the response time series and other psychological measures will indicate a common generating process of the multifractality of these signals. As an example, intermittent changes in attention and error correction could be verified by multifractal analyses of gaze fixation and eye movements during the same cognitive task (e.g., Kelty-Stephen and Mirman, 2013).

In summary, caution should be made when inferring response time series as multifractal in a strict mathematical sense. Nevertheless, the width of the multifractal spectra could still be a sensitive index of the intermittency of the response time series even though the intermittency is not prototypical multifractal. The main advantage of multifractal analyses of response time series is their ability to assess the temporal changes in their scale invariant structure. Further studies should focus on the assessment of generating processes of multifractal by experimentation under strong inference. This might include the assessment of temporal changes in the local scaling exponent (i.e., the local structure of response time variation) in more heterogeneous and real-life experiments where the task conditions and characteristics of the stimuli involve change across trials. Furthermore, the correlation between the temporal changes in the structure of the response time variation and other neurophysiological and psychological measurements can be assessed through multifractal analyses by correlating the temporal change of the scaling exponents (see example in Figure 7 in Ihlen and Vereijken, 2013). Time series from different levels of the cognitive and neurophysiological system are more likely to correlate in their scale independent structure rather than their unit dependent magnitude. Thus, multifractal analyses might provide new insight into the interaction and coordination of multiple levels of cognitive performance and human behavior.

### Conflict of interest statement

The author declares that the research was conducted in the absence of any commercial or financial relationships that could be construed as a potential conflict of interest.
